# Bibliometric Evaluation of the 100 Top-Cited Articles on Anesthesiology

**DOI:** 10.7759/cureus.50959

**Published:** 2023-12-22

**Authors:** Rakan Khalid Alfouzan, Pillai Arun Gopinathan, Ikram UI Haq, Kiran Iyer, Azzam Abdullaziz Nawab, Abdullah Alhumaidan

**Affiliations:** 1 Department of Anesthesiology, King Abdulaziz Medical City, Ministry of National Guard Health Affairs, Riyadh, SAU; 2 Department of Maxillofacial Surgery and Diagnostic Sciences, College of Dentistry, King Saud Bin Abdulaziz University for Health Sciences, Ministry of National Guard Health Affairs, Riyadh, SAU; 3 Department of Maxillofacial Surgery and Diagnostic Sciences, King Abdullah International Medical Research Centre, Riyadh, SAU; 4 College of Dentistry, King Saud Bin Abdulaziz University for Health Sciences, Riyadh, SAU; 5 Department of Preventive Dental Sciences, College of Dentistry, King Saud Bin Abdulaziz University for Health Sciences, Riyadh, SAU; 6 Department of Anesthesiology, Imam Mohammad Ibn Saud Islamic University, Riyadh, SAU; 7 Department of Medicine and Surgery, Imam Mohammad Ibn Saud Islamic University, Riyadh, SAU

**Keywords:** vosviewer, research productivity, citations, bibliometric analysis, anesthesia

## Abstract

This review is a bibliometric analysis based on anesthesiology, which is a medical specialty that deals with a patient's complete preoperative, intraoperative, and postoperative care. The objective of the review attempts to analyze the bibliometric characteristics of the 100 most top-cited articles on anesthesiology. The meta-data of the study were collected from the Core Collection of Web of Science database. A title search option was employed, and “Anesthesia” and “Anesthesiology” were typed in two different search boxes separated with the Boolean operator ''OR''. Further, the data were sorted by highest citation order; later, “article” was selected from the filter of document type, and all other types of documents were excluded. Finally, downloaded the bibliographic details of the 100 top-cited articles. VOSviewer Software (version 1.6.10 by van Eck and Waltman) was used for bibliometric network analysis for co-authors and keywords. Pearson chi-square test was used for statistical analysis.

The 100 top-cited articles were published between the years of 1971 and 2018. These articles gained a maximum of 1006 to a minimum of 276 citations with an average of 384.57 cites/article. Open accessed articles gained a slightly higher ratio of citations, while more than half of the articles were published in the two leading journals of “Anesthesiology” and “Anesthesia and Analgesia”. There was no statistically significant difference in both citation analysis among open and closed access journals and Anesthesia vs Non-Anesthesia journals. Thirty-six articles were published in journals not specifically related to Anesthesia. Most of the top-cited articles were contributed by the United States, whereas Surgery and General Anesthesia were the two most occurred keywords.

We conclude that all the top-cited articles in anesthesiology were contributed by authors who belonged to the developed nations and the United States outclassed the rest of the world. This bibliometric analysis would be valuable to practitioners, academics, researchers, and students to understand the dynamics of progress in the field of anesthesiology.

## Introduction and background

Anesthesiology, a prominent field in the health sciences, is concerned with patient care, which includes minimal discomfort and considering patient security before, during, and following any surgical intervention [1]. The agony of surgery was eradicated in 1846 via the successful application of anesthesia, which was considered one of the greatest horrors of humanity. Becoming pain-free must be among the very few medical milestones and accomplishments that can affect every person [2]. The first journal, the American Journal of Anesthesia and Analgesia, dedicated to anesthesia was published as a quarterly supplement to the American Journal of Surgery in 1914. Later, a bimonthly journal, Current Researches in Anesthesia and Analgesia (now Anesthesia and Analgesia), started in 1922, and the quarterly supplement was terminated in 1926 [3]. Since then, a number of journals and research publications have been published around the globe, and a notable increase in the scientific literature on anesthesia has been observed [4].

It is necessary to assess the growth of publications and their features periodically to re-evaluate the research priorities [5]. A bibliometric research approach has been used to quantify the prominent characteristics of publications and popular trends in research. The use of statistics and mathematics in scholarly literature has been known as bibliometric analysis [6,7]. Since the development of the internet and the availability of electronic databases such as PubMed, Web of Science, Scopus, and Google Scholar, bibliometric studies have acquired recognition within the academic world [8]. Research policies, financial allocations, and strategic decisions have been influenced by the findings of bibliometric studies. The quality of the research and the impact of its citations are crucial metrics for agencies to rank countries and institutions [9,10]. Academic progression decisions in the discipline of anesthesiology are informed by publication activity [11]. 

Literature shows bibliometric studies were performed in the field of anesthesiology. A recent study stated that 207,683 documents had been indexed under Anesthesiology and Pain Medicine in the Scopus database, and these documents covered the period from 1932 to 2022. About 41% of the documents were published during the last five years of study, while 39% of the documents were produced in the United States. Anesthesia and Analgesia, Regional Anesthesia and Pain Medicine, and Anesthesiology were the top three publication sources [11]. A study carried out the bibliometric analysis and measured the quantity of research on anesthesia, pain, critical care, and emergency medicine that had been published in 30 different journals between 1996 and 1997. The United States (40.2%) and the United Kingdom (13.3%) produced the greatest amount of research. Subsequently, these two countries (United States=17; United Kingdom=8) published most of the journals on anesthesia [12]. Dogan and Karaca reviewed anesthesia-related publications that were indexed in the Web of Science between 2009 and 2018. The United States produced more than one-fourth (28.9%) of the literature, with 89.7% of the documents published in English [4]. Another study measured the publication growth of anesthesiology from 1999 to 2018 in two different datasets. Comparatively, a low number of papers (n=69,593) was found in the subject dataset, while the department dataset had more than double papers (167,501), but their citation impact was slightly similar with 21.52 and 22.27 cites/doc, respectively [13]. A PubMed-based study reported that 6,736 clinical research studies on anesthesia were published between 2000 and 2005. Anesthesia and analgesia had the most studies published (18.92%), and 20% of the research contributed was from the United States. The study distributed the publications by country-wise income categories: high-income countries produced 45%, followed by upper-income countries with 25%, and low-income countries contributed 19% and 11% of the total publications, respectively [14].

A five-year (2004-2008) bibliometric evaluation of the 104 anesthesia researchers belonging to 23 academic institutions in the United Kingdom reported the four most productive institutions (Imperial College, Oxford, Cambridge and University College London), which produced 51% of the research and gained 54% of the total citations [15]. Xie et al. analyzed a 10-year research output of Chinese authors that was published in ten top-ranked journals of anesthesia, and China contributed 3.07% of the global anesthesia research that was related to basic and non-clinical varieties [16]. Another study reported that China, Taiwan, and Hong Kong region contributed 2.3% of the anesthesia research at the global level, while Taiwan published more research as compared to China [17]. Al-Fouzan and Haq inspected the publication growth of Saudi Arabia in Anesthesiology and Pain Management, which revealed that 1,085 documents were published from 1983 to 2022, and about 55% of documents were published during the last five years of the study (2018-2022). The Saudi Journal of Anesthesia was found to be the preferred source of publication, while Egypt and the United States were the top research-collaborating countries [11].

Buyukcoban et al. carried out a bibliometric study on the 100 most cited articles on geriatric anesthesia, which were published from 1980 to 2014, while 58 articles were published from 2000 to 2009. Most of the articles (21%) were published in Anesthesiology journal, and 50% of the articles were produced in the United States [18]. It is imperative to investigate the publication characteristics of the most influential articles on anesthesiology to understand the prevailing trends and patterns of research. The aim of the current review was to examine the bibliometric feathers of anesthesiology of the top 100 cited articles indexed in the Web of Science database.

## Review

Methods and search strategy

A bibliometric research method was employed to evaluate the bibliometric properties of the 100 top-cited articles on “Anesthesiology”. Since the study was retrospective, ethical clearance from the institutional review board was exempted. The data was obtained from the Web of Science database's Core Collection on November 12, 2023. The inclusion criteria were original study and review articles, while case reports, conference papers, brief surveys, books with notes, theses, comments and letters were excluded from the study.

The words “Anesthesia” OR “Anesthesiology” were inserted in the search box, and the option of “Title” instead of “All Field” was selected. Further, the selected “Article” from the document type filter was used. The data was sorted by highest citation order, and the bibliographic details of 100 top-cited articles were downloaded in Microsoft Excel (Microsoft Corporation, New York, USA) and plain text was used for data analysis. The examination of the periodic growth of articles, citations, frequently used journals, contributing countries, prolific authors, and most occurring keywords was presented in tabular and graphic formats. Microsoft Excel (V-16) and VOSviewer Software (version 1.6.10 by van Eck and Waltman) were used to analyze the data.

Statistical analysis

The 100 top-cited articles were analyzed to compare articles with citations published in open access and closed access journals. The citations were also compared between anesthesia journals and non-anesthesia journals. The Pearson chi-square test was used for the statistical analysis. The data was analyzed with SPSS Statistics for Windows, Version 20, 2011 (IBM Corp, Armonk, New York, USA). The p-value (<0.05) was considered statistically significant.

Results

**Figure 1:**
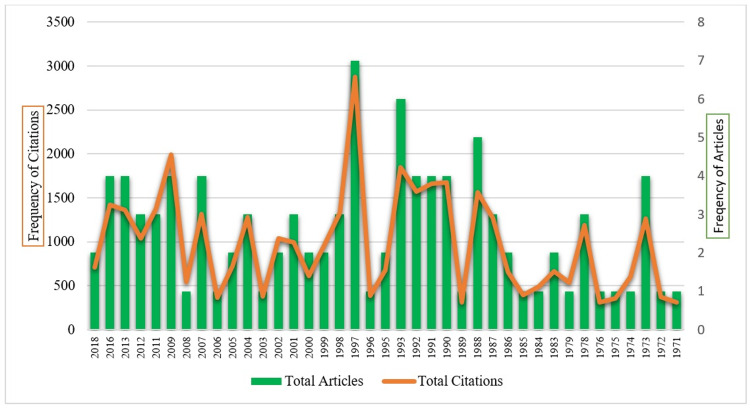
The top 100 most cited papers on anesthesiology by year-wise distribution. Note: This image is the author's own creation.

The 100 top-cited articles were published over a period of 48 years, from 1971 to 2018. The span of 48 years was divided into four intervals, with 12 years in each interval. The lowest number of articles (n=13) were published during the first interval (1971-1982), while one-third of articles (n=33) were published in the second interval (1983-1994), followed by 29 articles in the third interval (1995-2006). The highest number of top-cited articles (n=7) were published in the year 1997, followed by five articles in the year 1993 (Figure 1). These 100 top-cited articles gained 38,457 citations, with a mean ratio of 384.57 cites/article. The articles published in the third interval (1995-2006) gained the maximum citation ratio (400.89 cites/article).

The analysis of accessibility modes elaborates that 55 top-cited articles had been published in open access format, while 45 articles were in close access format. The open accessed articles received a higher ratio of citations (395.21 cites/article) as compared to the closed-accessed articles (371.55 cites/article). However, there was no statistically significant difference (p=0.39) in citations between open and closed access journals when the Pearson chi-square value was tested.

The 100 top-cited articles were published in 37 journals, while 28 journals published one article each. The maximum number of articles (n=37) were published in the journal Anesthesiology. The coverage span of these articles was 48 years, and the citation impact was 400.43 cites/article. The second most frequent source of publication was Anesthesia and Analgesia (n=19) with a coverage period of 39 years. More than half of the articles (n=56) were published in these two journals, while the remaining 44 papers were published in the other 35 journals. Table 1 shows the top journal distribution.

**Table 1:** Breakdown of topmost 100 cited papers by journal-wise distribution.

Name of journal	Impact factor (quartile)	Total articles	Coverage period	Total citations	Citations impact
Anesthesiology	8.8 (Q-1)	37	1971-2018	14,816	400.43
Anesthesia and Analgesia	5.9 (Q-1)	19	1973-2011	7,356	387.16
JAMA–Journal of American Medical Association	120.7 (Q-1)	4	1972-2016	1,526	381.50
British Journal of Anesthesia	9.8 (Q-1)	2	1988-1993	634	317.00
Journal of Neuroscience	5.3 (Q-1)	2	1997-2007	631	315.50
Journal of Neurosurgical Anesthesiology	3.7 (Q-2)	2	2009-2013	805	402.50
Neurosurgery	4.8 (Q-1)	2	1993-2000	658	329.00
New England Journal of Medicine	158.5 (Q-1)	2	1992-2008	1,040	520.00
Pediatrics	8.0 (Q-1)	2	2011-2012	1,027	513.50
Pain	7.4 (Q-1)	1	1979	537	537.00

About one-third of the articles (n=64) were published in the eight journals related to the category of anesthesia, and these articles gained an average of 394.21 cites/article. The remaining 36 articles were published in 29 other journals not specifically related to anesthesia, and these articles gained a slightly lesser citation impact (367.41 cites/article). However, there was no statistically significant difference (p=0.36) in citation between anesthesia journals and non-anesthesia journals when the Pearson chi-square value was tested.

The New England Journal of Medicine had the highest impact factor, followed by the Journal of American Medical Association (JAMA). Both of these journals accepted quality articles on all categories of biomedical sciences. Among the journals related to anesthesia, the British Journal of Anesthesia had the maximum impact factor, followed by Anesthesiology. Out of the top-10 journals, nine journals were in the high quartile (Q-1) scale range. 

Table 2 shows the distribution of articles by country-wise, which elaborates on the authors belonging to 16 countries who contributed to the 100 top-cited articles on anesthesiology. Authors affiliated with the United States contributed the maximum number of articles (n=60), followed by France and Sweden with seven and six articles, respectively. Two countries (Canada and England) contributed five articles each. Although Switzerland was positioned in the 10th rank with one article, this article gained the maximum citation impact (584 cites/article), followed by Israel (463 cites/paper).

**Table 2:** Distribution of papers and citations by contributing countries.

Rank	Country	Total articles	Total citations	Citation impact
1.	United States	60	23,303	388.38
2.	France	7	3,123	446.14
3.	Sweden	6	1,944	324.00
4.	Canada	5	2,087	417.40
5.	England	5	1,714	342.80
6.	Germany	4	1,434	358.50
7.	Italy	3	1,040	346.67
8.	Israel	2	926	463.00
9.	Australia	2	765	382.50
10.	Switzerland	1	584	584.00
11.	People's Republic China	1	436	436.00
12.	Belgium	1	425	425.00
13.	South Korea	1	354	354.00
14.	Spain	1	350	350.00
15.	Federal Republic Germany	1	348	348.00
16.	Ireland	1	305	305.00

A total of 528 authors contributed to the 100 top-cited articles, and 489 (92.61%) authors contributed to one article each. A small number of authors (n=39; 7.39%) had published more than one article. The details of top-15 productive authors are shown in Table 3. Darrell R. Schroeder and Robert R. Caplan emerged as the top two prolific authors with five papers each, but based on citation impact, Darrell R. Schroeder was the top-ranked author. Among the top 15 authors, 13 belonged to the United States and one each from France and Sweden. Overall, Yves Auroy was positioned in the 12th rank and his articles gained the maximum citation impact (552 cites/article), followed by a group of five authors (Randall P. Flick, Slavica K. Katusic, Juraj Sprung, David O. Warner, and Robert T. Wilder) with 549.25 cites/article.

**Table 3:** Productive authors with affiliation, total articles, total citations, and citation impact.

Ranks	Author’s name	Affiliation	Total articles	Total citations	Citation impact
1.	Darrell R. Schroeder	Mayo Clinic, Rochester, Minnesota, United States	5	2,565	513.00
2.	Robert A. Caplan	Virginia Mason Medical Center, Seattle, United States	5	1,834	366.80
3.	Randall P. Flick	Mayo Clinic, Rochester, Minnesota, United States	4	2,197	549.25
4.	Slavica K. Katusic	Mayo Clinic, Rochester, Minnesota, United States	4	2,197	549.25
5.	Juraj Sprung	Mayo Clinic, Rochester, Minnesota, United States	4	2,197	549.25
6.	David O. Warner	Mayo Clinic, Rochester, Minnesota, United States	4	2,197	549.25
7.	Robert T. Wilder	Mayo Clinic, Rochester, Minnesota, United States	4	2,197	549.25
8.	Guohua Li	Columbia University, New York, United States	4	1,696	424.00
9.	Lena S. Sun	Columbia University Medical Center, New York, United States	4	1,696	424.00
10.	Charles Dimaggio	Mailman School of Public Health, New York, United States	4	1696	424.00
11.	Karen L. Posner	University of Washington, Seattle, United States	4	1570	392.50
12.	Yves Auroy	Service d’Anesthésie-Réanimation, Hôpital d’Instruction des Armées Percy, Clamart, France	3	1656	552.00
13.	Karen B. Domino	University of Washington, Seattle, United States	3	1,066	355.33
14.	Frederick W. Cheney	Committee of Professional Liability, American Society of Anesthesiologists, United States	3	1,222	407.33
15.	Goran Hedenstierna	Huddinge University Hospital, Sweden	3	966	322.00

**Figure 2:**
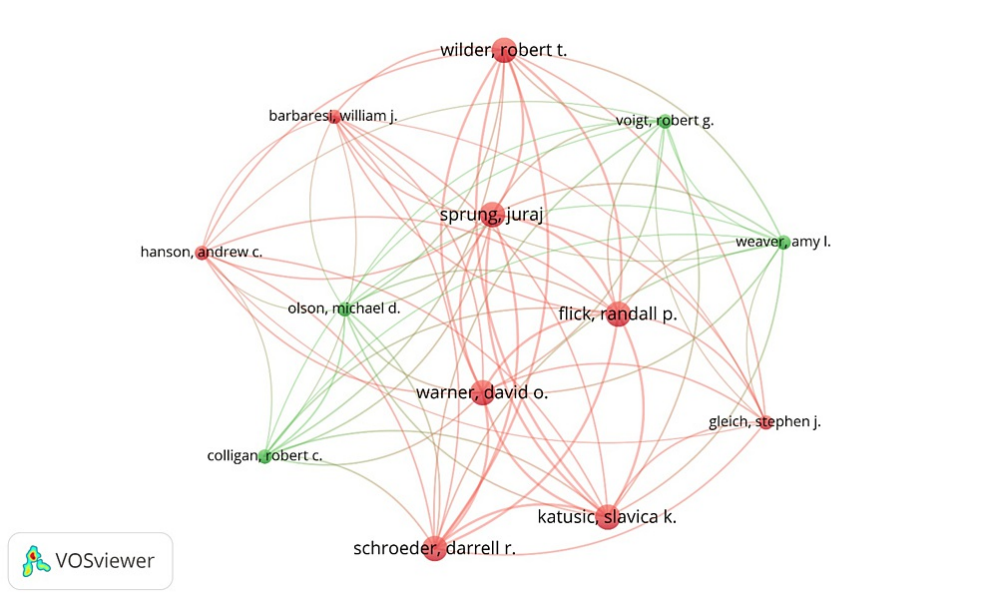
Research co-authorship among different authors by Vosviewer. Note: This image is the author's own creation.

**Figure 3:**
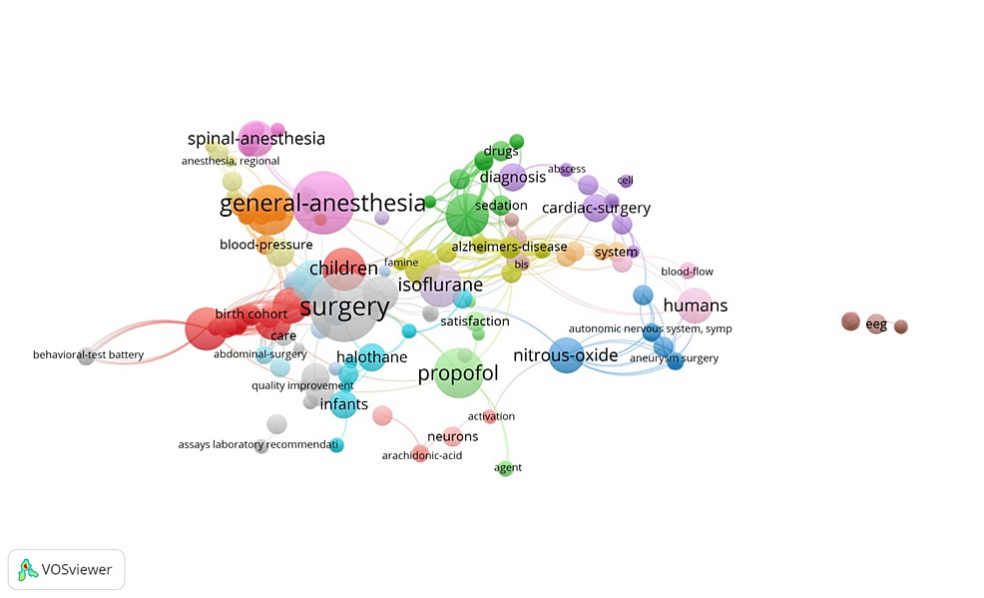
Keyword co-occurrence network map visualization by Vosviewer. Note: This image is the author's own creation.

Out of 39 top productive authors, 13 authors had connected in two clusters of co-authorship networks generated by VOSviewer Software (Figure 2). In the first cluster, nine authors (William J. Barbarest, Randall P. Flick, Stephen J. Gleich, Andrew C. Hanson, Slavica K. Katusic, Darrell R. Schroeder, Juraj Sprung, David O. Warner, and Robert T. Wilder), while in the second cluster, only four authors (Robert C. Colligan, Michael D. Olson, Robert G. Volgt, and Amy L. Weaver) have been connected with each other. The VOSviewer software was also used to assess the co-occurrence rates of keywords. A total of 520 keywords were identified, and 474 keywords were connected with each other as shown in Figure 3. The top 26 keywords with occurrence rates ranging from a maximum of nine to a minimum of three times have been provided in Table 4. The keyword of surgery has occurred the highest number of times (n=9), followed by general anesthesia (n=8), bupivacaine and propofol with six times each.

**Table 4:** Most common keywords.

Rank	Keywords	Occurrence
1.	Surgery	Nine times
2.	General anesthesia	Eight times
3.	Bupivacaine and propofol	Six times each
4.	Anesthesia; apoptotic neurodegeneration; children; isoflurane; risk	Five times each
5.	Association; cardiac-arrest; humans; ketamine; learning-disabilities; nitrous-oxide; risk-factors; spinal-anesthesia	Four times each
6.	Brain; cardiac-surgery; diagnosis; halothane; infants; management; mortality; neurotoxicity; outcomes	Three times each

Appendix 1 (Table 5) shows the detailed bibliographic details of the 100 most cited articles, among which the most influential article was ''Early Exposure to Anesthesia and Learning Disabilities in a Population-Based Birth Cohort,’' which received the most citations (1006). However, as per the evaluation of annual citation density, the article entitled ‘'Regional Anesthesia in the Patient Receiving Antithrombotic or Thrombolytic Therapy: American Society of Regional Anesthesia and Pain Medicine Evidence-Based Guidelines (Fourth Edition)’' was the most dynamic publication (70.83 citation density).

Discussion

The findings of the present bibliometric review are noteworthy and offer research scholars inclusive scrutiny of the 100 top-cited articles on anesthesiology as represented in the Web of Science database. Significant research has been identified by its nature of work as well as by measuring the number of citations. Citation metrics recognize the method to assess the qualitative aspects of research publications [19,20]. 

In the present review, 100 top-cited articles on anesthesiology were published in 48 years from 1971 to 2018, while 62 articles were published in the middle years from 1983 to 2006. Another study on 100 top-cited articles on propofol-related infusion syndrome revealed that these articles were published from 2001 to 2023, while most of the articles (n=65) were published in the middle period from 2009 to 2015 [21]. Tripathi et al. examined the bibliometric features of 2,519 H-classic articles on anesthesiology, which were published from 1945 to 2008; the maximum number of articles was published between 1980 and 1990 [22]. Another study reported that 207,683 documents were indexed under Anesthesiology and Pain Medicine in Scopus from 1932 to 2022, and about 41% of the documents were published in the last five years from 2018 to 2022 [11]. A study examined 151 papers on anesthesia, which were produced by King Fahd Hospital of Saudi Arabia over a span of 30 years (1983 to 2013), of which 60% of the papers were published in the first 24 years and 40% in the last six years [23].

In our study, 100 top-cited articles were published in 37 journals in six countries. A maximum of 27 journals were published in the United States, followed by the United Kingdom with six journals and one each from Canada, Denmark, Egypt, and the Netherlands. Eighty-nine articles were published in 27 journals published in the United States, and the remaining 11 articles were published in another 10 journals published in five countries. Fifty-eight articles published in three anesthesiology journals gained higher average citations (396.15 cites/article) as compared to 31 articles published in non-anesthesiology journals (368.41 cites/article). The journal Anesthesiology was found most frequent (n=37) with a maximum coverage span and citation impact of 400.43 cites/article, followed by Anesthesia and Analgesia (n=19). More than half of the articles were published in these two journals. The anesthesiology journals had 64 articles, which gained an average of 394.21 cites/article, while the other 36 articles published in non-anesthesiology journals gained a lower citation impact (367.41 cites/article). Tripathi et al. stated that 72% of the H-index articles on anesthesia were published in anesthesiology, while 28% were published in non-anesthesiology journals, followed by Pain Journal with 22% [22]. Another study summarized the global research on anesthesia from 1932 to 2022, which reported that Anesthesia and Analgesia, Regional Anesthesia and Pain Medicine, and Anesthesiology were the top three publication sources [11]. In our review, 36% of articles were published in non-anesthesiology journals, and more than half (n=56) of the articles were published in two journals, Anesthesiology and Anesthesia and Analgesia. Another bibliometric study focused on geriatric anesthesia reported that more than three-fourths of the articles were published in journals published from the United States, followed by the United Kingdom [18]. A bibliometric study on Pediatric Anesthesia stated Pediatric Anesthesia was found to be the most frequent source of publications, followed by Anesthesia and Analgesia and Anesthesiology. These top three journals were published in the United States [24].

Most of the studies supported the fact that the highest number of studies in anesthesiology have been produced in the United States. A Scopus-based study from 1932 to 2022 revealed that 39% of the documents were produced in the United States [11]. Another bibliometric study focused on Pediatric Anesthesia stated that 39% of the literature was produced in the United States, followed by Canada with 6.3% [24]. A study of 100 top-cited articles on propofol-related infusion syndrome revealed that the United States contributed about half (n=49) of the articles [21]. Further bibliometric analysis of anesthesia was restricted to the dataset of two years from 2007 to 2008, which reported that 89% of the literature had been produced by high-income nations and the rest of the world produced nominal literature [25]. In line with these statistics, our study also endorsed that the United States contributed the highest number of top-cited articles (n=60) on anesthesiology, followed by France and Sweden with seven and six articles, respectively. The reason could be due to the fact that developed nations have achieved notable positions in scientific research and have established research-producing institutions along with flourishing research cultures long ago. Most of the developing countries have focused on scholarship and research during the last quarter of the 20th century and their research productivity has increased during the last two decades [26-28].

The article contributed by Wilder et al. on “Early Exposure to Anesthesia and Learning Disabilities in a Population-Based Birth Cohort” got the highest number of citations (n=1006), but gained second position according to analysis of citation density by year-wise distribution [29]. With regard to citation density by year, the article contributed by Horlocker et al. entitled “Regional Anesthesia in the Patient Receiving Antithrombotic or Thrombolytic Therapy: American Society of Regional Anesthesia and Pain Medicine Evidence-Based Guidelines” gained the first position overall; however, this article conceded the 23rd position with 425 citations [30].

Analyzing the findings of our review showed that 55% of the most cited articles were published in an open access format, and these articles gained a better citation impact as compared to subscription-based articles. Another bibliometric study focused on anesthesiology research in Saudi Arabia reported that 58.50% of the research was published in open access format, and the ratio of open access increased during the 21st century [11]. The present study also stated that among the top 15 productive authors, 13 belonged to the United States. Darrell R. Schroeder of the Mayo Clinic emerged as the most influential author, followed by Robert A. Caplan. As per the Web of Science author profile, Darrell R. Schroeder contributed 513 documents in 40 years from 1984 to 2023, with an average of 13 documents per year, and his research work was cited 21,258 times with a mean ratio of 41.43 cites/doc. Whereas Robert A. Caplan had 76 documents indexed, which were cited 10,958 times with an average of 144.18 cites/doc. The co-occurrence networks of keywords were performed with the word “Anesthesia,” which was found to be the most common keyword, followed by “Pain,” “Analgesia,” and “COVID-19”. In the future, researchers can conduct a thorough analysis of subject dispersion to identify both the strengths and weaknesses of anesthesiology and pain medicine research.

The findings in the present study would serve as a yardstick for upcoming studies on anesthesiology. Most of the top-cited research was contributed by the authors of developed nations, along with collaboration in quality research performance. The stakeholders of developing countries, such as government agencies, health ministries, academic institutions, research centers, and associations of anesthesiology, should come to step forward and start research collaboration on novel and innovative ideas to develop the art and science of anesthesiology.

Limitations

The current study is limited to the analysis of the Web of Science database, which could lead to the elimination of important articles that Web of Science doesn't index. Other databases, like Scopus, PubMed, and Google Scholar, have different scopes and coverages. Although the Scopus database has comprehensive coverage, the Web of Science claims to provide quality literature [31]. Google Scholar has its limitations, as it does not offer the affiliation search query. Future studies may retrieve the dataset from other databases and conduct a comparative study. Another limitation was the keyword 'Anesthesia' which was used in the search query and was restricted to standard American (US English) vocabulary. Future studies may use alternative spellings to expand the scope of research. There is also a need for the inclusion of more journals on anesthesiology, and currently, 65 journals are indexed in the Web of Science database. The inclusion of more journals from multiple databases would add to the impact of journals and published articles for comprehensive coverage of such a vast topic, thus increasing the visibility and volume of anesthesiology research.

## Conclusions

Periodically conducting qualitative analysis is essential to assessing the current research trends in anesthesiology. The results support the development of expertise and the establishment of new research standards. The authors from 16 countries contributed to the top 100 top-cited articles on anesthesiology. The United States outclassed the high-income developed countries and contributed the highest number of top-cited articles. The outcomes illustrate that there is a strong correlation between economic growth and quality research productivity. The governments of these countries took gigantic initiatives to develop a strong foundation in medical education, research, and healthcare delivery systems a long time ago, and the results of these efforts are vividly clear.

## Appendices

Appendix 1 

**Table 5:** Bibliographic details of 100 top-cited articles on anesthesiology.

Serial no.	Title of articles	Citations	Citation density by year (rank)
1	Early exposure to anesthesia and learning disabilities in a population-based birth cohort [29]	1006	67.07 (2)
2	Serious complications related to regional anesthesia: results of a prospective survey in France [32]	688	25.48 (17)
3	Epidural anesthesia and analgesia in high-risk surgical patients [33]	648	17.51 (37)
4	Adverse respiratory events in anesthesia: a closed claims analysis [34]	624	18.35 (35)
5	Which clinical anesthesia outcomes are important to avoid? The perspective of patients [35]	615	24.60 (21)
6	Major complications of regional anesthesia in France: The SOS Regional Anesthesia Hotline Service [36]	606	27.55 (16)
7	Effects of anesthesia and paralysis on diaphragmatic mechanics in man [37]	604	12.08 (61)
8	Cognitive and behavioral outcomes after early exposure to anesthesia and surgery (published correction appears in Pediatrics) [38]	601	46.23 (5)
9	Learning manual skills in anesthesiology: Is there a recommended number of cases for anesthetic procedures? [39]	584	22.46 (25)
10	Preventable anesthesia mishaps: a study of human factors [40]	568	12.35 (60)
11	Anesthesia awareness and the bispectral index [41]	543	33.94 (11)
12	Autotomy following peripheral nerve lesions: experimental anesthesia dolorosa [42]	537	11.93 (62)
13	Association between a single general anesthesia exposure before age 36 months and neurocognitive outcomes in later childhood [43]	517	64.63 (3)
14	Halothane-morphine compared with high-dose sufentanil for anesthesia and postoperative analgesia in neonatal cardiac surgery [44]	497	15.53 (43)
15	An analysis of major errors and equipment failures in anesthesia management: considerations for prevention and detection [45]	494	12.35 (59)
16	Development and psychometric evaluation of the pediatric anesthesia emergence delirium scale [46]	468	23.40 (23)
17	The tissue origin of low back pain and sciatica: a report of pain response to tissue stimulation during operations on the lumbar spine using local anesthesia [47]	459	13.91 (49)
18	Bispectral index monitoring allows faster emergence and improved recovery from propofol, alfentanil, and nitrous oxide anesthesia. BIS Utility Study Group [48]	457	16.93 (39)
19	Cauda equina syndrome after continuous spinal anesthesia [49]	448	13.58 (50)
20	The sedative component of anesthesia is mediated by GABA(A) receptors in an endogenous sleep pathway [50]	437	19.86 (31)
21	CODA Trial Group. BIS-guided anesthesia decreases postoperative delirium and cognitive decline [51]	436	39.64 (7)
22	Long-term differences in language and cognitive function after childhood exposure to anesthesia [52]	426	35.50 (10)
23	Regional anesthesia in the patient receiving antithrombotic or thrombolytic therapy [30]	425	70.83 (1)
24	Anesthesia-related deaths during obstetric delivery in the United States [53]	422	15.63 (41)
25	Anesthesia crisis resource management training: teaching anesthesiologists to handle critical incidents [54]	416	13.00 (52)
26	Effects of epidural anesthesia and analgesia on coagulation and outcome after major vascular surgery [55]	411	12.45 (57)
27	The incidence of awareness during anesthesia: a multicenter United States study [56]	408	20.40 (28)
28	TREK-1, a K+ channel involved in neuroprotection and general anesthesia [57]	400	20.00 (30)
29	Pulmonary densities during anesthesia with muscular relaxation--a proposal of atelectasis [58]	398	10.21 (73)
30	The lateral pressure profile in membranes: a physical mechanism of general anesthesia [59]	396	14.67 (45)
31	Ketamine anesthesia during the first week of life can cause long-lasting cognitive deficits in rhesus monkeys [60]	395	30.38 (12)
32	Neurological complications after regional anesthesia: contemporary estimates of risk [61]	392	23.06 (24)
33	Epidemiology and morbidity of regional anesthesia in children: a one-year prospective survey of the French-Language Society of Pediatric Aneasthesiologist [62]	391	13.96 (48)
34	Postoperative pain after inguinal herniorrhaphy with different types of anesthesia [63]	389	11.44 (64)
35	Reinfarction following anesthesia in patients with myocardial infarction [64]	387	9.44 (78)
36	Early childhood exposure to anesthesia and risk of developmental and behavioral disorders in a sibling birth cohort [65]	384	29.54 (14)
37	Groupe d'Etudes des Réactions Anaphylactoïdes Peranesthésiques. Anaphylactic and anaphylactoid reactions occurring during anesthesia in France in 1999-2000 [66]	378	18.00 (36)
38	Perioperative outcomes of major hepatic resections under low central venous pressure anesthesia: blood loss, blood transfusion, and the risk of postoperative renal dysfunction [67]	376	14.46 (46)
39	The effects of body mass on lung volumes, respiratory mechanics, and gas exchange during general anesthesia [68]	373	14.35 (47)
40	Predictors of hypotension after induction of general anesthesia [69]	372	19.58 (32)
41	Myocardial infarction after general anesthesia [70]	370	7.12 (91)
42	A retrospective cohort study of the association of anesthesia and hernia repair surgery with behavioral and developmental disorders in young children [71]	369	24.60 (22)
43	Heat flow and distribution during induction of general anesthesia [72]	368	12.69 (54)
44	Survey of anesthesia-related mortality in France [73]	362	20.11 (29)
45	A comparison of endoscopic ultrasound, magnetic resonance imaging, and exam under anesthesia for evaluation of Crohn's perianal fistulas [74]	359	15.61 (42)
46	Olsson GL, Hallen B, Hambraeus-Jonzon K. Aspiration during anaesthesia: a computer-aided study of 185,358 anaesthetics [75]	359	9.45 (77)
47	Molecular connectivity. I: Relationship to nonspecific local anesthesia [76]	359	7.33 (90)
48	Modification of cortical stimulation for motor evoked potentials under general anesthesia: technical description [77]	355	11.45 (63)
49	Anesthesia induces neuronal cell death in the developing rat brain via the intrinsic and extrinsic apoptotic pathways [78]	354	18.63 (34)
50	Pharmacokinetics and pharmacodynamics of propofol infusions during general anesthesia [79]	351	9.75 (75)
51	EEG complexity as a measure of depth of anesthesia for patients [80]	350	15.22 (44)
52	Tumescent technique for regional anesthesia permits lidocaine doses of 35 mg/kg for liposuction [81]	349	10.26 (70)
53	Anesthesia-related cardiac arrest in children: update from the Pediatric Perioperative Cardiac Arrest Registry [82]	348	20.47 (27)
54	Randomised trial of fentanyl anaesthesia in preterm babies undergoing surgery: effects on the stress response [83]	348	9.41 (79)
55	One hundred percent incidence of hemidiaphragmatic paresis associated with interscalene brachial plexus anesthesia as diagnosed by ultrasonography [84]	345	10.45 (68)
56	Haemodynamic changes during anaesthesia induced and maintained with propofol [85]	345	9.58 (76)
57	Development and psychometric testing of a quality of recovery score after general anesthesia and surgery in adults [86]	339	13.56 (51)
58	Perioperative morbidity in patients randomized to epidural or general anesthesia for lower extremity vascular surgery. Perioperative Ischemia Randomized Anesthesia Trial Study Group [87]	338	10.90 (66)
59	The inguinal paravascular technic of lumbar plexus anesthesia: the "3-in-1 block" [88]	338	6.63 (94)
60	Effects of regional anesthesia on phantom limb pain are mirrored in changes in cortical reorganization [89]	336	12.44 (58)
61	The milk-ejection reflex of the rat: an intermittent function not abolished by surgical levels of anaesthesia [90]	333	6.53 (96)
62	Protective mechanical ventilation during general anesthesia for open abdominal surgery improves postoperative pulmonary function [91]	331	30.09 (13)
63	Incidence and risk factors for side effects of spinal anesthesia [92]	329	10.28 (69)
64	Sympathetic responses to induction of anesthesia in humans with propofol or etomidate [93]	327	10.22 (72)
65	The incidence and nature of adverse events during pediatric sedation/anesthesia with propofol for procedures outside the operating room: a report from the Pediatric Sedation Research Consortium [94]	323	21.53 (26)
66	Myocardial reinfarction after anesthesia and surgery [95]	321	6.98 (92)
67	Effect of conscious sedation vs general anesthesia on early neurological improvement among patients with ischemic stroke undergoing endovascular thrombectomy: a randomized clinical trial [96]	318	39.75 (6)
68	Differences in short-term complications between spinal and general anesthesia for primary total knee arthroplasty [97]	318	28.91 (15)
69	Pediatric anesthesia morbidity and mortality in the perioperative period [98]	316	9.29 (81)
70	Reproductive outcome after anesthesia and operation during pregnancy: a registry study of 5405 cases [99]	316	9.03 (84)
71	Cerebral protection by barbiturate anesthesia. Use after middle cerebral artery occlusion in Java monkeys [100]	313	6.52 (97)
72	Anesthesia, pregnancy, and miscarriage: a study of operating room nurses and anesthetists [101]	311	5.87 (99)
73	Anesthesia-related cardiac arrest in children: initial findings of the Pediatric Perioperative Cardiac Arrest (POCA) Registry [102]	310	12.92 (53)
74	Dynamic and static cerebral autoregulation during isoflurane, desflurane, and propofol anesthesia [103]	309	10.66 (67)
75	The pressure reversal of general anesthesia and the critical volume hypothesis [104]	308	6.04 (98)
76	Low- and high-frequency membrane potential oscillations during theta activity in CA1 and CA3 pyramidal neurons of the rat hippocampus under ketamine-xylazine anesthesia [105]	307	9.90 (74)
77	Hospital stay and mortality are increased in patients having a "triple low" of low blood pressure, low bispectral index, and low minimum alveolar concentration of volatile anesthesia [106]	305	25.42 (18)
78	Attention-deficit/hyperactivity disorder after early exposure to procedures requiring general anesthesia [107]	305	25.42 (19)
79	Anesthetic requirements and cardiovascular effects of fentanyl-oxygen and fentanyl-diazepam-oxygen anesthesia in man [108]	304	6.61 (95)
80	Intracerebral microdialysis in clinical practice: baseline values for chemical markers during wakefulness, anesthesia, and neurosurgery [109]	303	12.63 (55)
81	Comparison of cuffed and uncuffed endotracheal tubes in young children during general anesthesia [110]	300	11.11 (65)
82	Complications associated with anaesthesia-a prospective survey in France [111]	298	7.84 (88)
83	Practice guidelines for obstetric anesthesia: an updated report by the American Society of Anesthesiologists Task Force on Obstetric Anesthesia and the Society for Obstetric Anesthesia and Perinatology [112]	296	37.00 (8)
84	Anesthesia leads to tau hyperphosphorylation through inhibition of phosphatase activity by hypothermia [113]	295	17.35 (38)
85	A comprehensive anesthesia simulation environment: re-creating the operating room for research and training [114]	295	8.19 (85)
86	Fish sedation, analgesia, anesthesia, and euthanasia: considerations, methods, and types of drugs [115]	293	19.53 (33)
87	Effect of epidural anesthesia and analgesia on perioperative outcome: a randomized, controlled veterans affairs cooperative study [116]	289	12.57 (56)
88	Re-expansion of atelectasis during general anaesthesia: a computed tomography study [117]	289	9.32 (80)
89	Long-term survival for patients undergoing volatile versus IV anesthesia for cancer surgery: a retrospective analysis [118]	288	36.00 (9)
90	Unexpected cardiac arrest during spinal anesthesia: a closed claims analysis of predisposing factors [119]	288	8.00 (86)
91	Neuropsychological and behavioral outcomes after exposure of young children to procedures requiring general anesthesia: The Mayo Anesthesia Safety in Kids (MASK) Study [120]	285	47.50 (4)
92	Development of an anesthesiology-based postoperative pain management service [121]	283	7.86 (87)
93	Effects of anesthesia and muscle paralysis on respiratory mechanics in normal man [122]	283	5.55 (100)
94	Tumescent technique for local anesthesia improves safety in large-volume liposuction [123]	282	9.10 (82)
95	Advances in and limitations of up-and-down methodology: a précis of clinical use, study design, and dose estimation in anesthesia research [124]	280	16.47 (40)
96	Comparison of rocuronium, succinylcholine, and vecuronium for rapid-sequence induction of anesthesia in adult patients [125]	280	9.03 (83)
97	Lung collapse and gas exchange during general anesthesia: effects of spontaneous breathing, muscle paralysis, and positive end-expiratory pressure [126]	279	7.54 (89)
98	Selective anesthesia-induced neuroinflammation in developing mouse brain and cognitive impairment [127]	277	25.18 (20)
99	Titration of volatile anesthetics using bispectral index facilitates recovery after ambulatory anesthesia [128]	276	10.22 (71)
100	Thromboembolism after total hip replacement: role of epidural and general anesthesia [129]	276	6.73 (93)
